# Identification of Lysine Succinylome and Acetylome in the Vancomycin-Intermediate Staphylococcus aureus XN108

**DOI:** 10.1128/spectrum.03481-22

**Published:** 2022-11-14

**Authors:** Li Tan, Yi Yang, Weilong Shang, Zhen Hu, Huagang Peng, Shu Li, Xiaomei Hu, Xiancai Rao

**Affiliations:** a Department of Microbiology, College of Basic Medical Sciences, Key Laboratory of Microbial Engineering Under the Educational Committee in Chongqing, Army Medical University (Third Military Medical University), Chongqing, China; University of Florida; Shanghai Pulmonary Hospital, School of Medicine, Tongji University; Southwest University

**Keywords:** posttranslational modification, *Staphylococcus aureus*, VISA, succinylation, acetylation, SaCobB

## Abstract

Protein posttranslational modifications (PTMs) play important roles in regulating numerous biological functions of prokaryotic and eukaryotic organisms. Lysine succinylation (Ksucc) and acetylation (Kac) are two important PTMs that have been identified in various bacterial species. However, the biological functions of Ksucc and Kac in vancomycin-intermediate S. aureus (VISA) remain unclear. In this study, we systematically identified 3,260 Ksucc sites in 799 proteins and 7,935 Kac sites across 1,710 proteins in the VISA strain XN108. Functional analyses revealed that both Ksucc and Kac sites were highly enriched in several critical metabolic pathways, including ribosomal metabolism, tricarboxylic acid cycle, and glycolysis. Furthermore, a remarkable cross talk between Ksucc and Kac modifications was observed that almost 75% of the succinylated sites were also frequently acetylated. In addition, we identified SaCobB, a Sirtuin 2-like lysine deacetylase, as a bifunctional enzyme with both deacetylation and desuccinylation activities in S. aureus. We demonstrated the first lysine succinylome and acetylome in a VISA and identified SaCobB, a functional enzyme taking part in the regulation of Ksucc and Kac in S. aureus. Our findings provide valuable information for further study on the regulatory mechanisms of PTMs in S. aureus.

**IMPORTANCE** Lysine succinylation (Ksucc) and acetylation (Kac) are two important protein posttranslational modifications (PTMs) that regulate numerous biological functions in prokaryotes and eukaryotes. However, the functions of Ksucc and Kac in Staphylococcus aureus are seldom described. Understanding of Ksucc and Kac modifications in S. aureus will facilitate the development of new strategies to control infections. Herein, we quantified both Ksucc and Kac in a vancomycin-intermediate S. aureus (VISA) strain XN108, analyzed the interaction between these two PTMs, and identified SaCobB as a bifunctional enzyme with both deacetylation and desuccinylation activities. This study is the first description of dual PTMs, Ksucc and Kac profiles, in the VISA. The findings could provide valuable information for the following researches on the regulatory roles of PTMs in S. aureus.

## INTRODUCTION

The classic central dogma states that DNA, as the main carrier of genetic information, is the major determinant of life science. Recently, increasing evidence has emphasized the importance of proteins, since they are the actual executors of most biological activities (1, 2). Generally, the translated precursor proteins are not always biologically active, and most of them require a series of processes, which is well known as posttranslational modifications (PTMs), to become mature proteins with biological functions (3, 4). The PTMs are divided into proteolytic cleavage and chemical modification, while the latter one attracts a lot more attention due to the existence of a variety of chemical PTM types. To date, many chemical PTMs have been characterized in diverse organisms. Classical PTMs such as phosphorylation (5), glycosylation (6, 7), and acetylation (8, 9) have been well investigated. These PTMs play important roles in regulation of all aspects of biological events, including signal transduction, cellular biological process, inflammation initiation, and disease progression (10, 11). The development of mass spectrometry (MS) technology enables the discovery of many novel PTMs, including succinylation, propionylation, malonylation, butyrylation, benzoylation, hydroxybutyrylation, and glutarylation (12–14), and has greatly benefited the studies on protein modifications.

Lysine acetylation (Kac) is one of the most well studied PTMs and extensively exists in eukaryotes and prokaryotes (15). Acetylation profiles and the related acetyltransferases have been identified in many bacteria (16). Studies have shown that acetylation plays an important role in regulating bacterial virulence and drug resistance (17, 18). Lysine succinylation (Ksucc) is a new type of PTM that was first identified in Escherichia coli in 2011 (19). Then, Ksucc modifications in other microorganisms, including Histoplasma capsulae (19), Vibrio parahaemolyticus (19), Porphyromonas gingivalis (20), Aeromonas hydrophila (21), and Mycobacterium tuberculosis (22), have been described. The global protein succinylation is reported to be closely associated with numerous important cellular processes, such as glycolysis, citrate cycle (trichloroacetic acid [TCA] cycle), oxidative phosphorylation, and protein biosynthesis (23). As two different types of PTMs, both Kac and Ksucc take place at lysine residues. Thus, a high probability of cross talk might exist between these PTMs. Zhou et al. performed an multiple PTM analysis in Candida albicans and demonstrated that different modifications can occur at the same site, and coordinately affect biological processes (24). Nevertheless, similar studies are extremely insufficient. Little is known about the roles of a target site modified by diverse PTMs, and the internal regulatory mechanisms require elucidation.

Staphylococcus aureus is an important human pathogen that distributes widely in hospitals and in communities (25, 26). Nevertheless, the work of PTM identification was seldom carried out in S. aureus. Only Zhang et al. (27) and Campbell et al. (28) detected the protein acetylation and succinylation in S. aureus before. In the present study, we conducted systematic analyses of both Ksucc and Kac modifications in S. aureus XN108, the first ST239 vancomycin-intermediate S. aureus (VISA) strain isolated in China (29). Up to 3,260 Ksucc sites in 799 proteins, and 7,935 Kac sites in 1,710 proteins were identified. The characterization of succinylome and acetylome in XN108 revealed a frequent cross talk between Kac and Ksucc modifications. In addition, we identified a sirtuin-like SaCobB protein as a bifunctional enzyme with desuccinylase and deacetylase activities in XN108. To our knowledge, this is the first description of dual PTMs, the succinylation and acetylation profiles, in VISA.

## RESULTS

### Profiles of Ksucc and Kac modifications in VISA strain XN108.

**FIG 1:**
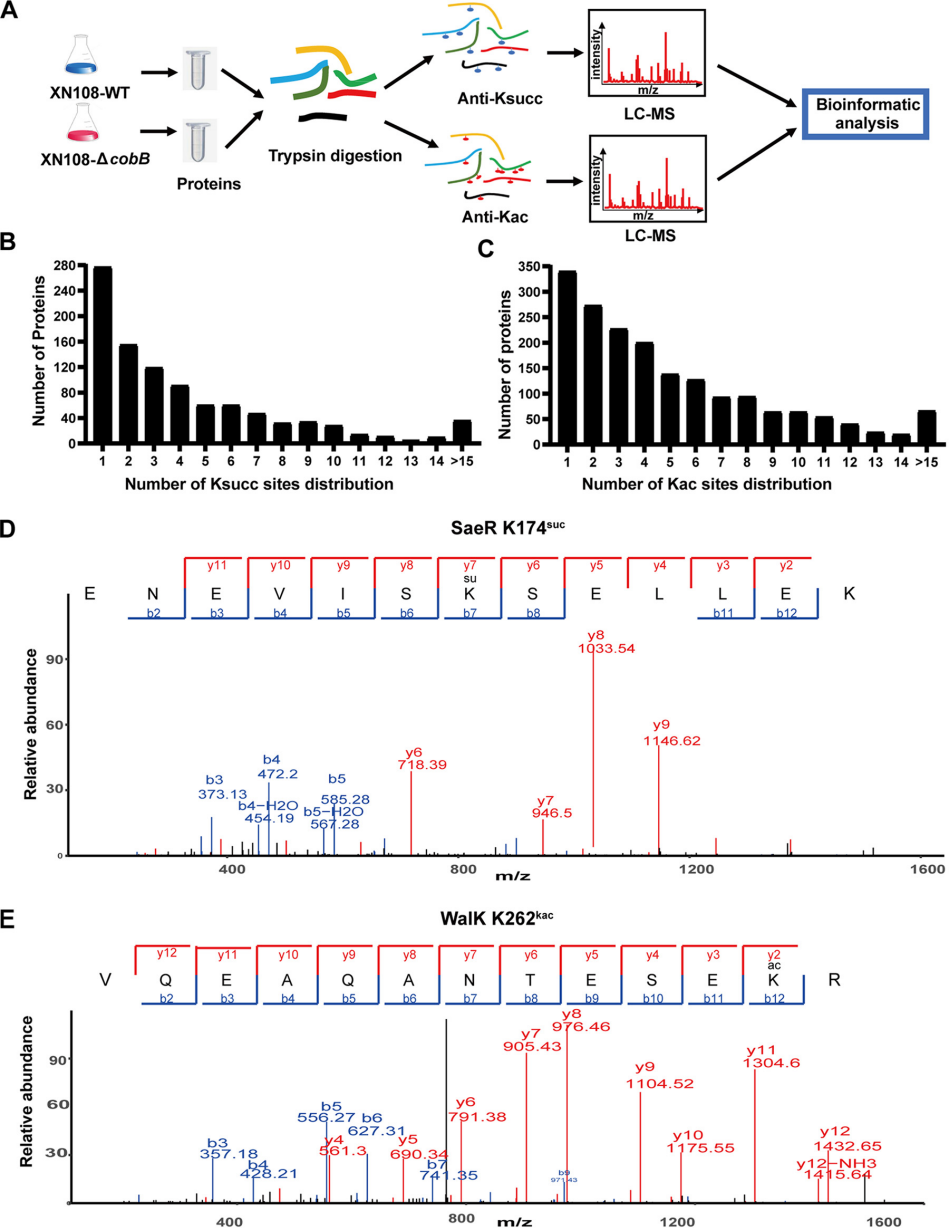
Label-free quantitative analysis of lysine succinylome and acetylome in vancomycin-intermediate S. aureus (VISA) XN108. (A) Workflow for identifying and quantifying the lysine succinylome or acetylome of VISA XN108. Proteins from the wild-type (WT) strain and Δ*cobB* mutant (*n *= 3) were digested with trypsin and spiked with succinylated or acetylated peptide standard, respectively. The enrichment peptides were analyzed by liquid chromatography tandem mass spectrometry (LC-MS). (B, C) Distribution of the succinylated sites (B) and acetylated sites (C) in proteins of XN108 were counted and indicated. (D) Representative MS/MS spectra of succinylated -ENEVISKsuccSELLEK- with a succinylated site at K174 from the protein SaeR (AID39215.1). (E) Representative MS/MS spectra of acetylated -VQEAQANTESEKacR- with an acetylated site at K262 from the protein WalK (AID38462.1).

The total protein sample of S. aureus strain XN108 was prepared and digested with trypsin to detect protein succinylation and acetylation in VISA. Then, the modified peptides were enriched with corresponding specific antibodies and were characterized by liquid chromatography tandem MS (Fig. 1A). All of the raw data were submitted to the ProteomeXchange data set (accession number PXD033341).

A total of 3,260 succinylation sites on 799 proteins were identified in XN108 wild-type strain (details listed in Data Set S1). Among the identified Ksucc proteins, about 90% were succinylated at 1 or 2 sites, the remaining proteins were succinylated at no less than 3 sites, including 12 proteins containing more than 20 Ksucc sites (Fig. 1B). To verify the specificity of succinylation sites in the MS characterization, an immunoprecipitation (IP) experiment was performed to pull down MurA protein, and its succinylated form was confirmed by using mouse anti-succinyllysine monoclonal antibody (MAb) (Fig. S1). As for acetylation modification, a total of 7,935 Kac sites on 1,710 proteins were identified (Data Set S2), among which 35.8% proteins were acetylated at 1 or 2 sites, the remaining proteins were acetylated at no fewer than 3 sites, and 47 proteins had at least 20 Kac sites (Fig. 1C). The identified succinylated and acetylated proteins accounted for 26.7% (799 of 2,992) and 57.2% (1,710 of 2,992) of the total proteins in XN108, respectively. Fig. 1D and 1E showed the representative mass spectra of succinylated and acetylated proteins, respectively. These results indicated that both Ksucc and Kac modifications were widespread in VISA strain XN108, and the range of Kac modification was much more abundant.

**FIG 2:**
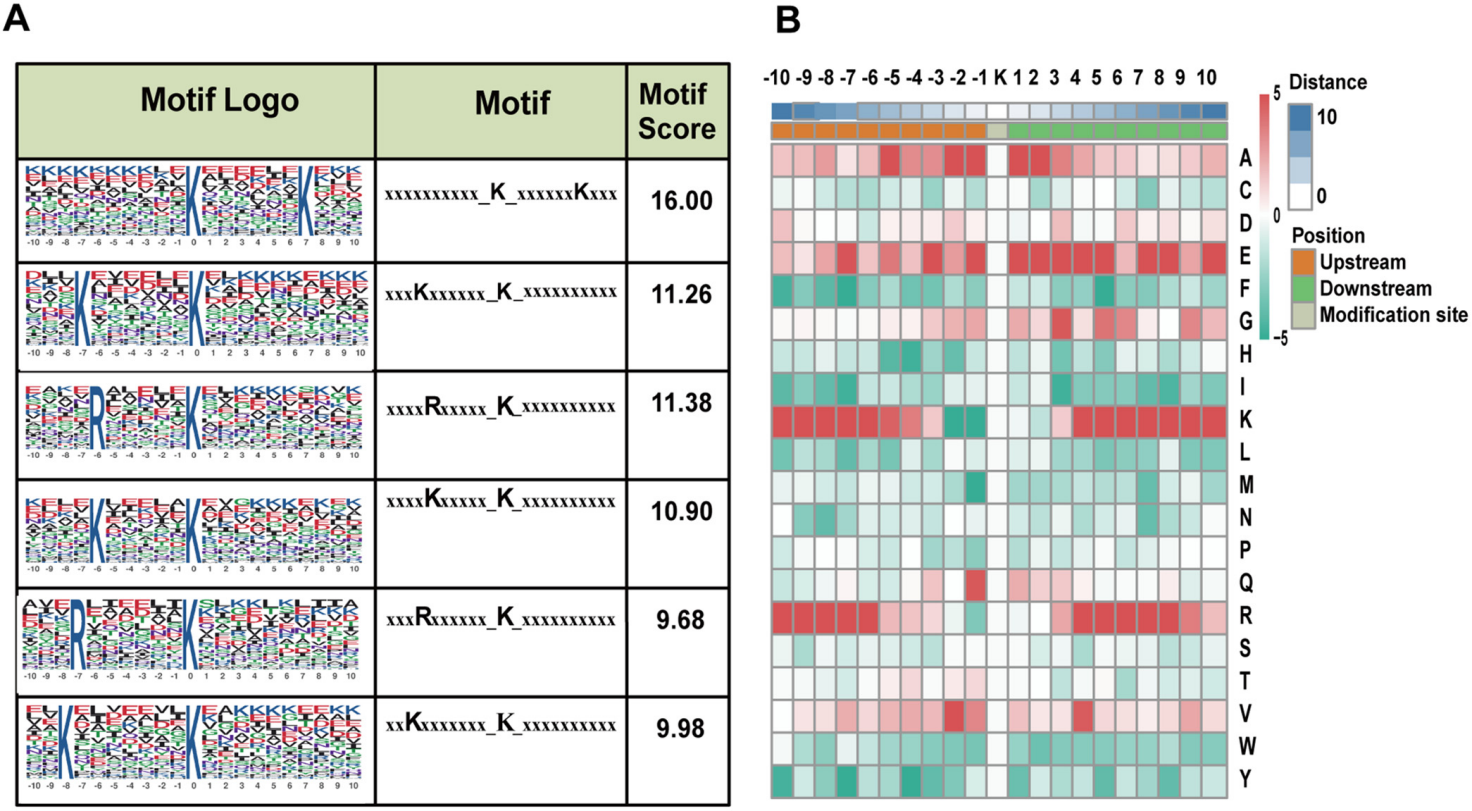
Motif analysis of identified lysine-succinylated sequences. (A) Ksucc sequences were analyzed using the Motif-x tool. The top six lysine succinylation motifs are shown. (B) Heat map showing the frequency of different amino acid residues around succinylated lysine residues in S. aureus.

The Motif-X program was applied to search for the overrepresented sequence patterns of all identified succinylated or acetylated peptides. For succinylome, the most representative consensus sequence motifs were KsuccXXXXXXK, KXXXXXXKsucc, RXXXXXKsucc, KXXXXXKsucc, RXXXXXXKsucc, and KXXXXXXXKsucc (where X indicates a random amino acid residue) (Fig. 2A). Among these motifs, the KXXXXXKsucc pattern is previously reported in Mycobacterium tuberculosis and Pseudomonas aeruginosa (22, 30), and the KXXXXXXKsucc and KXXXXXXXKsucc motifs are found in P. aeruginosa and rice seeding (30, 31). The acetylation motifs were quite distinct from the succinylation ones, and the six most representative motifs were KacS, KacN, KacT, KacY, KacH, and Kac F (Fig. S2A). All of the six acetylation motifs are reported in other prokaryotes and eukaryotes, such as KacY and KacH in Vibrio parahemolyticus (32) and KacT, KacH, KacS, KacN, and KacF in human cells (33). In addition, the specific amino acids flanking the upstream and downstream of the Ksucc and Kac sites were also analyzed (Fig. 2B; Fig. S2B).These results indicated widespread of the mentioned sequence motifs among different species, including S. aureus.

### Functional annotation of lysine succinylome and acetylome in XN108.

**FIG 3:**
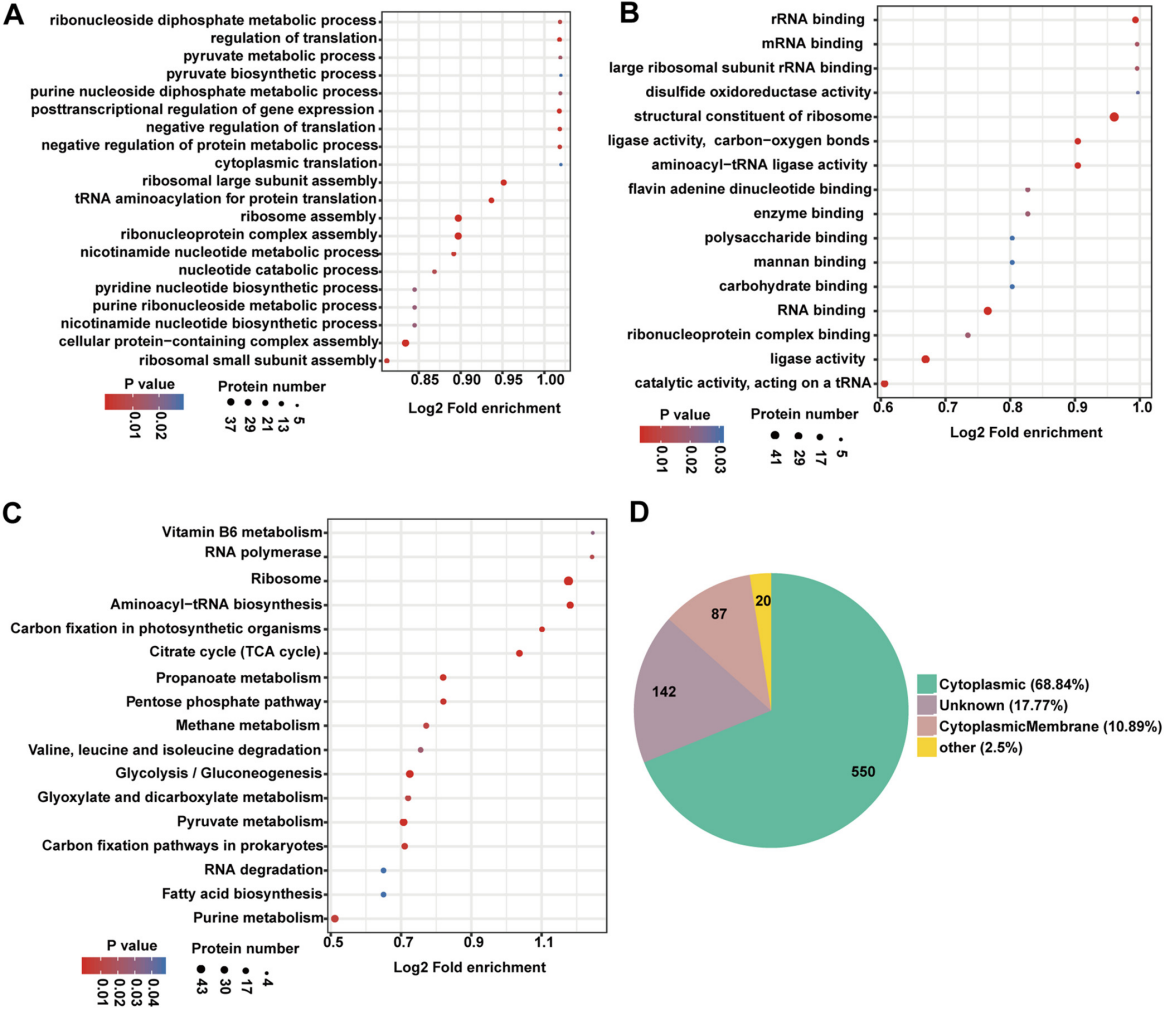
Gene Ontology (GO) enrichment and Kyoto Encyclopedia of Genes and Genomes (KEGG) pathway analysis of Ksucc proteins in VISA XN108. (A, B) GO analysis for the biological processes (A) and molecular function (B) of identified succinylated proteins in VISA XN108. (C) KEGG categories of succinylated proteins in VISA XN108. (D) Subcellular localization of the identified succinylated proteins from GO enrichment. TCA, trichloroacetic acid.

The Gene Ontology (GO) annotation and Kyoto Encyclopedia of Genes and Genomes (KEGG) analysis of all identified succinylated proteins were performed to understand the roles of Ksucc in VISA XN108. Bubble plots were constructed to visualize the proteins containing succinylation sites (Fig. 3). GO annotation analysis showed that the largest groups of succinylated proteins were mostly annotated to metabolic and cellular process, especially in ribosome and ribonucleoprotein complex assembly, cellular protein-containing complex assembly, and tRNA aminoacylation for protein translation (Fig. 3A). Based on molecular function classification, the succinylated proteins were mostly classified into structure constituents of ribosome and proteins with ligase activity, catalytic activity, or RNA binding ability (Fig. 3B). KEGG pathway analysis revealed that the succinylated proteins were mainly involved in pathways of microbial ribosome, glycolysis/gluconeogenesis, and pyruvate metabolism (Fig. 3C). Within the cluster of cellular localization, nearly 70% of succinylated proteins were enriched in cytoplasmic category, 10.89% were enriched in cytoplasmic membrane, and the remaining 17.77% succinylated proteins cannot be classified (Fig. 3D). The analysis results of acetylated proteins were almost the same as those of the succinylated proteins. GO analysis revealed that the acetylated proteins were mainly enriched in metabolic and cellular process, binding and catalytic activity, and cytoplasmic category (Fig. S3). KEGG analysis showed that the acetylated proteins involved in the pathways associated with ribosome, amino sugar and nucleotide sugar metabolism, and pyruvate and purine metabolism were highly enriched (Fig. S4). Overall, these data suggest that the cellular process of metabolism is highly correlated with protein succinylation and acetylation in the VISA strain XN108.

### Analysis of the cross talk between Ksucc and Kac modification in XN108.

**FIG 4:**
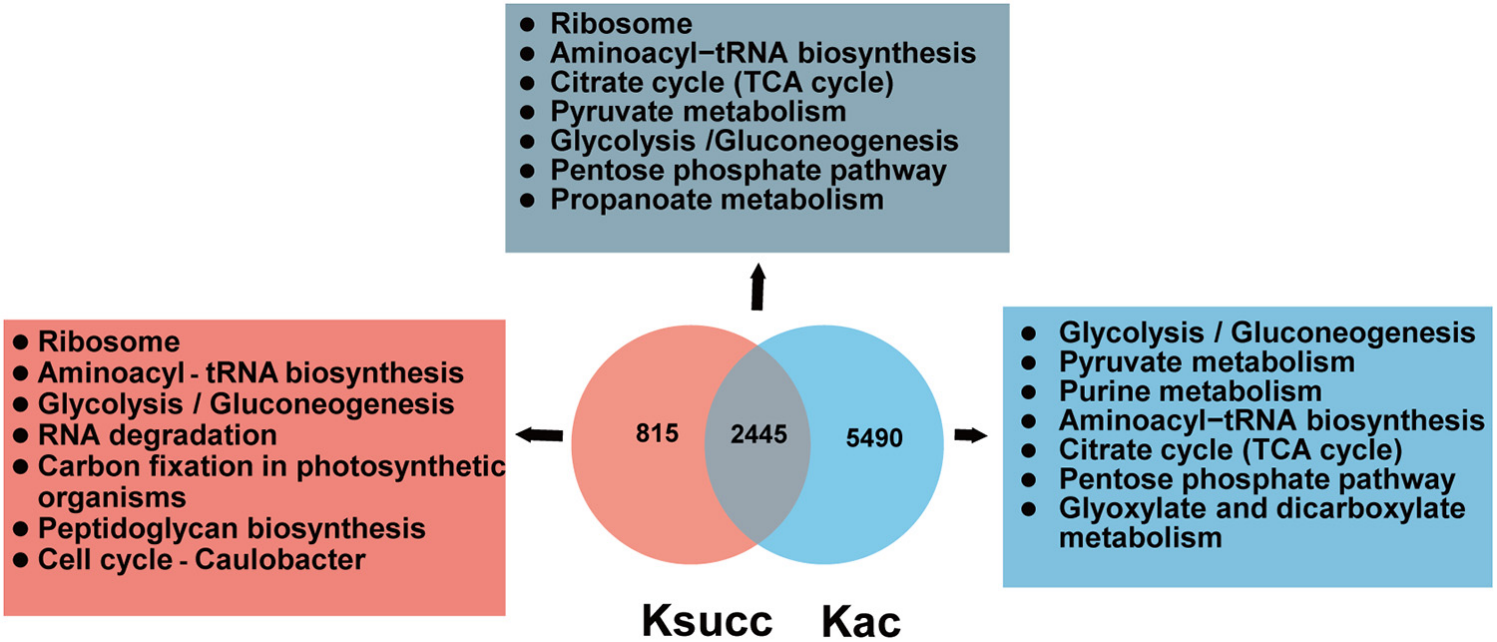
Cross talk between succinylation (Ksucc) and acetylation (Kac) in VISA XN108. Venn diagram shows the number of protein sites overlapping among succinylation and acetylation. The gray, pink, and blue areas indicate the KEGG pathways involved in the overlapping, unique succinylated, and unique acetylated proteins, respectively.

Both succinylation and acetylation occur at lysine, and previous studies suggest cross talk between Ksucc and Kac PTMs in several bacteria, including E. coli (34, 35), A. hydrophila (21), and P. aeruginosa (30). In this study, comparison of succinylome data with that of acetylome was performed to determine the extent of overlap between protein succinylation and acetylation in XN108. Among the total 3,260 Ksucc sites, up to 75% (2,445 of 3,260) sites were also frequently targets of acetylation, whereas only 25% (815 of 3,260) were unique for succinylation (Fig. 4; Data Set S3). This finding indicates that strong cross talk exists between protein succinylation and acetylation in S. aureus strain XN108.

To further understand the biological meanings of Ksucc and Kac dual-modified proteins, the GO classification was performed, and the results showed that the overlapped proteins were mainly enriched in central metabolism associated pathways, such as catalytic enzymes in the ribosome, aminoacyl-tRNA biosynthesis, and glycolysis/gluconeogenesis biosynthetic pathway (Fig. 4). Strikingly, 10 key enzymes involved in glycolysis/gluconeogenesis pathway were dual-modified. On the contrary, the succinylation alone was more likely to occur in proteins involved in RNA degradation, peptidoglycan biosynthesis, and bacterial cell cycle, whereas proteins related to glyoxylate and dicarboxylate metabolism were unique to acetylation. Our results suggest that the identified Ksucc and Kac modifications interact frequently in XN108 and might synergistically regulate the central metabolism of VISA.

### Characterization of SaCobB as an enzyme with desuccinylation and deacetylation bifunctions in XN108.

In the process of protein acylation and deacylation, enzymes are needed to catalyze the transfer of acyl groups to different amino acid residues. A study has reported that the Sir2-like enzyme CobB shows comparable deacetylase and desuccinylase bifunction in E. coli (34), whereas Streptomyces coelicolor uses two different sirtuin-like proteins to perform deacetylase and desuccinylase function, respectively (36). Specifically, ScCobB2 possesses desuccinylase activity, while ScCobB1 has deacetylase role in S. coelicolor (37). In 2019, Burckhardt and coworkers reported that a Sir2-like enzyme designated SaCobB exhibits deacetylase activity in S. aureus HG001 (38). By homologous BLAST analysis, we found a highly conserved protein named NAD-dependent protein deacetylase of the SIR2 family (locus_tag=SAXN108_2450, AID40891.1) in S. aureus XN108. Compared with SaCobB, AID40891.1 lacks only three amino acids at its N terminus. Therefore, we also designed AID40891.1 as SaCobB in this study. Next, we wondered whether SaCobB (AID40891.1) possesses only deacetylase activity or has both deacetylase and desuccinylase activities in S. aureus, like CobB in E. coli.

**FIG 5:**
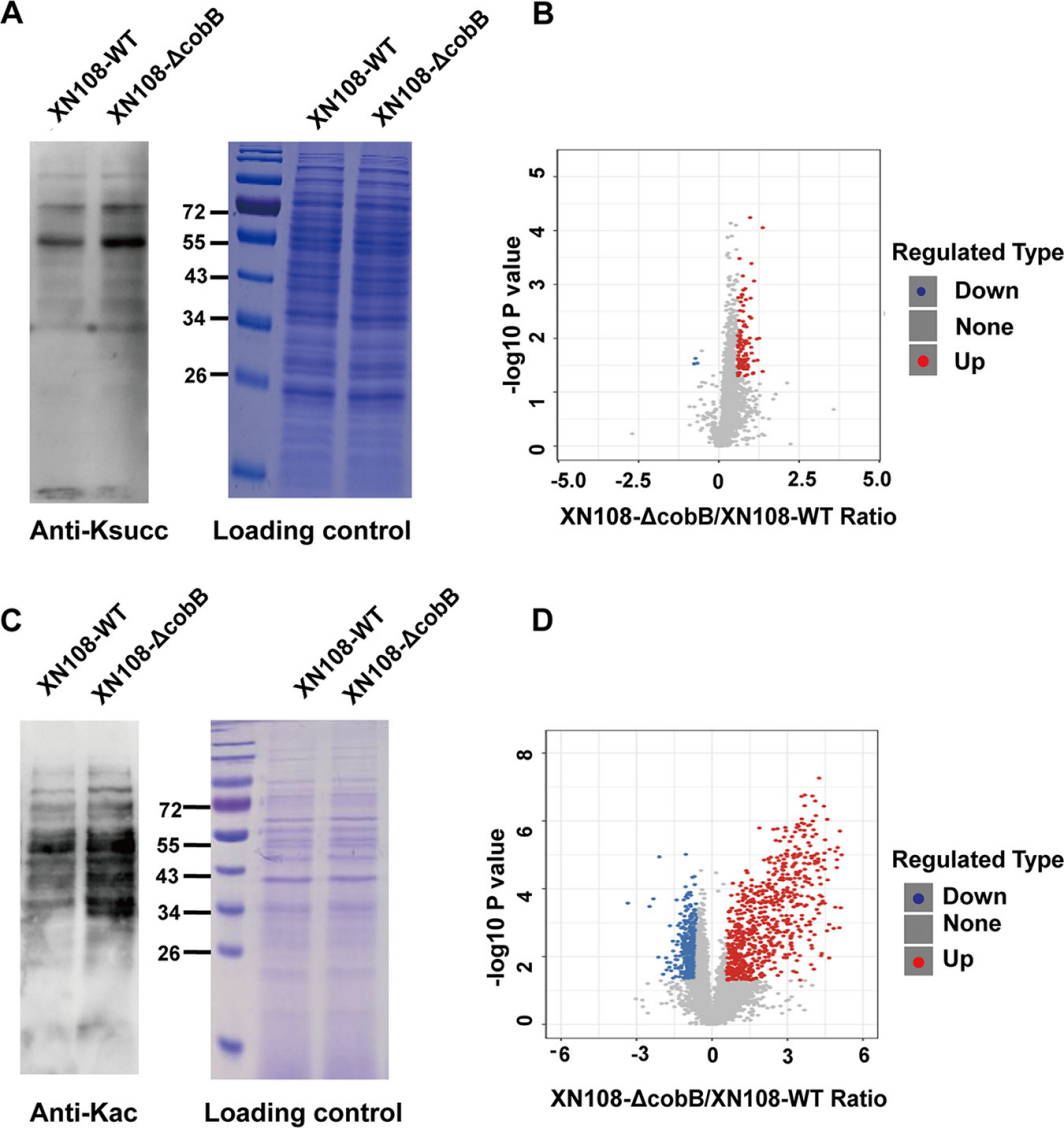
Characterization of SaCobB as a bifunctional desuccinylase and deacetylase in S. aureus XN108. (A, C) The global succinylation (A) and acetylation (C) levels of XN108-WT and XN108-Δ*cobB* were determined by Western blotting with pan-anti-succinyllysine and pan-anti-acetyllysine antibodies, respectively. Proteins stained with Coomassie blue were set as the loading control. (B, D) Volcano plot illustrated significantly differential abundant Ksucc (B) and Kac (D) proteins of the quantitative proteome analysis. The red dots indicate upregulated modified proteins, the green dots indicate downregulated modification proteins, and the gray dots show comparable modification proteins.

To determine the desuccinylase and deacetylase activity of SaCobB *in vivo*, the XN108-Δ*cobB* mutant was constructed (Fig. S5). Western blot was performed to detect the PTM levels of XN108-WT and XN108-Δ*cobB*. As shown in Fig. 5A and 5C, the protein band phenotypes between two strains were almost the same; nevertheless, both succinylation and acetylation levels of XN108-Δ*cobB* strain were slightly enhanced compared with that of XN108-WT, indicating that SaCobB might function as both a desuccinylase and a deacetylase *in vivo*.

Label-free-based quantitative lysine succinylome and acetylome analyses between XN108-Δ*cobB* and XN108-WT were performed using antibody-based affinity enrichment, followed by liquid chromatography-mass spectrometry (LC-MS/MS) analysis (Fig. 5). In order to ensure the high credibility of the results, each group was independently repeated three times, and the correlation of the protein intensity (log_10_ scale) among three biological replicates of the XN108-WT and XN108-Δ*cobB* mutant displayed a high correlation (Pearson's correlation >0.8; Fig. S6), suggesting a reliable quantification analysis. In addition, a standard of localization probability >0.75 was used to filter the identification data. For succinylome analysis, as many as 2,936 succinylation sites on 741 proteins were quantified and normalized to the proteome data (Data Set S4). With a quantification ratio of >1.5 (*P* value < 0.05) as upregulation threshold and <1/1.5 (*P* value < 0.05) as downregulation threshold, 101 Ksucc sites across 85 succinylated proteins were found to be regulated by SaCobB. Among which, 98 sites on 83 proteins were hypersuccinylated, and only 3 sites on 2 proteins were hyposuccinylated after *cobB* gene deletion as exhibited by volcano plot (Fig. 5B; Data Set S5). As for protein acetylation, after a similar process, 7,047 acetylation sites on 1,557 proteins in total were quantified and normalized to the proteome data (Data Set S6), and 1,281 Kac sites on 825 proteins were further detected to be influenced by SaCobB. Specifically, the acetylation level of 704 sites on 426 proteins were enhanced, whereas that of 577 sites on 399 proteins weakened in XN108-Δ*cobB* compared with those in the XN108-WT (Fig. 5D; Data Set S7). These results demonstrate that SaCobB regulates both protein succinylation and acetylation in VISA strain XN108.

### Functional annotation of SaCobB-regulated lysine succinylome and acetylome in XN108.

GO annotation and KEGG analysis of SaCobB-regulated Ksucc and Kac proteins were performed to make clear the regulatory role of SaCobB in S. aureus. The GO classification results for biological processes showed that the largest group of succinylated proteins regulated by SaCobB was annotated to metabolic and cellular process (26.60%), especially in purine nucleotide biosynthetic process and ribonucleoside-related biosynthetic process (Fig. S7A). Based on molecular function classification, SaCobB-affected proteins were mainly associated with peptidase activity (Fig. S7B). GO classification showed that SaCobB-regulated Kac-modified proteins were enriched in metabolic and cellular processes, including tRNA aminoacylation for protein translation, catalytic proteins, and cytoplasmic proteins (Fig. S8A, S8B, and S8D). KEGG analysis indicated that the proteins regulated by SaCobB were mainly involved in central metabolism and biosynthetic processes (Fig. S8C) and were particularly involved in pathways associated with carbon metabolism, pyruvate metabolism, and ribosome and microbial metabolism in diverse environments. Interestingly, we found that proteins associated with peptidoglycan synthesis have a high tendency to undergo succinylation or acetylation modification (Fig. S9A). Furthermore, several proteins associated with oxidative stress and bacterial survival and a few transcriptional regulators regulated by SaCobB were observed (Fig. S9B; Data Set S8). Overall, these results indicate that the proteins, of which the Ksucc and Kac modifications were regulated by SaCobB, are highly involved in central process of S. aureus metabolism and biosynthesis.

### Analysis of functional interactions of SaCobB-regulated succinylated and acetylated proteins.

**FIG 6:**
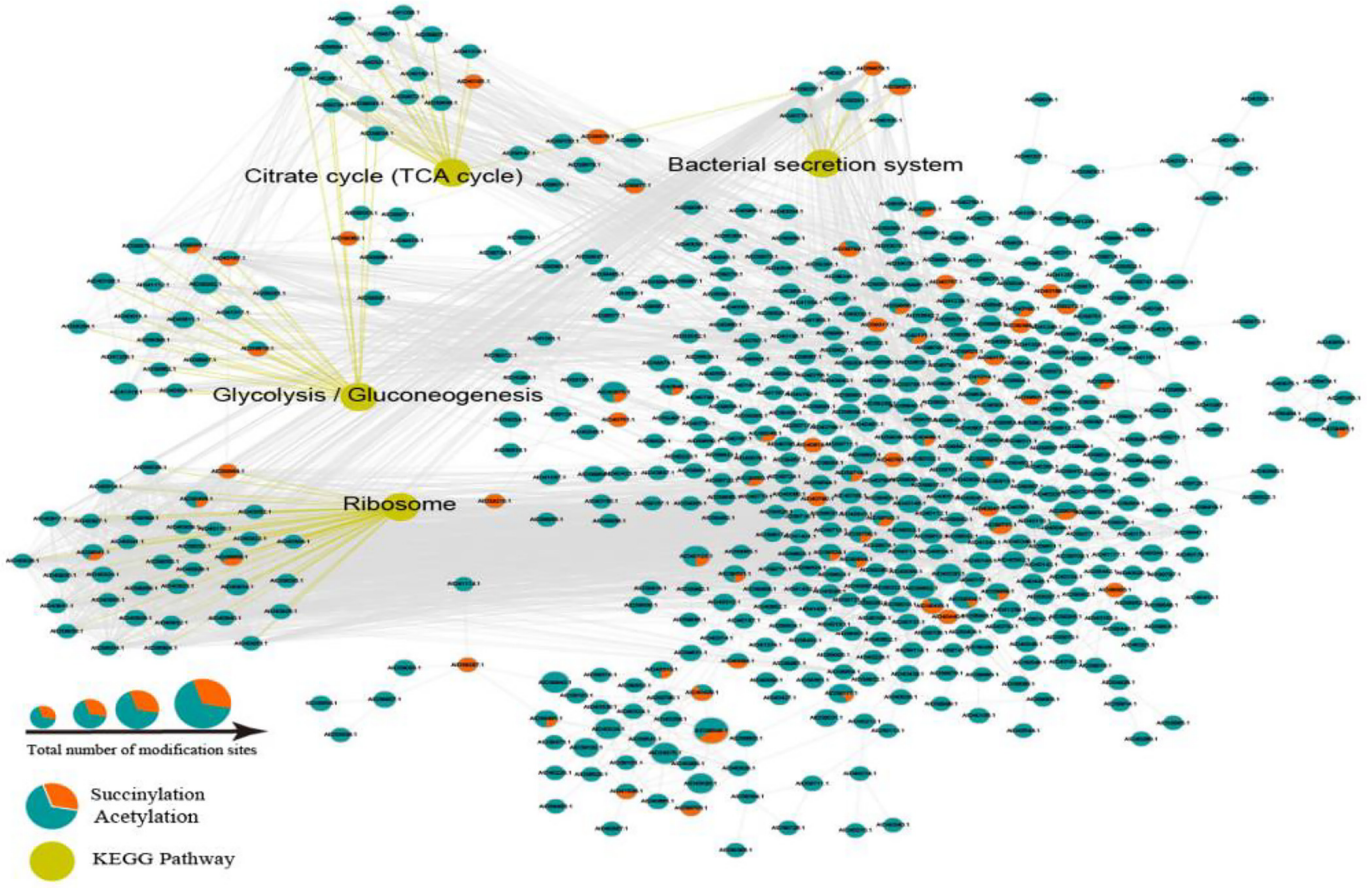
Protein-protein interaction networks in succinylated and acetylated proteins regulated by SaCobB. Four highly interconnected subnetworks in succinylated or acetylated proteins regulated by SaCobB are indicated. They are ribosome, TCA cycle, glycolysis/gluconeogenesis, and the bacterial secretion system. The circles in the diagram represent the differentially expressed proteins, and the orange and blue colored circles represent the different modification sites (orange for succinylation sites, blue for acetylation site). The yellow represent the Kyoto Encyclopedia of Genes and Genomes (KEGG) pathway. The protein IDs of the nodes in the network are shown in Data Set S9.

To understand the biological meaning of SaCobB-influenced proteins, all upregulated and downregulated succinylated and acetylated proteins in XN108-Δ*cobB* compared with those in the XN108-WT were subjected to protein-protein interaction (PPI) analysis based on the STRING database. Interactions identified with high confidence (confidence score >0.7) were mapped with STRING and visualized with Cytoscape software. Four highly interconnected networks containing SaCobB-regulated succinylated or acetylated proteins were indicated (Fig. 6). The first network consists of several proteins involved in ribosome process. The second network consists of some proteins involved in TCA central metabolism. The third cluster contains glycolysis/gluconeogenesis pathway-related proteins, and the last cluster contains modified proteins associated with the bacterial secretion system. However, since the number of SaCobB-affected Ksucc proteins is limited, such proteins were enriched only in the ribosome process-associated network (Fig. S10A). Meanwhile, SaCobB-regulated Kac proteins were clustered into all four networks (Fig. S10B). In addition, we found that proteins with upregulated Kac levels (marked in red) were more frequently distributed in ribosome network, whereas proteins with downregulated Kac levels (marked in blue) were relatively more common in glycolysis/gluconeogenesis network (Fig. S10B). Interestingly, proteins with unique acetylation widely existed in different PPI networks. On the contrary, proteins with unique succinylation did not exist in the glycolysis/gluconeogenesis pathway. Taken together, these results suggest that acetylation and succinylation regulated by SaCobB are pleiotropic in VISA XN108. Cooperation and competition among Ksucc and Kac protein complexes may play important roles in protein synthesis and bacterial metabolism in S. aureus.

## DISCUSSION

Identification of various kinds of PTMs in different species is of great importance. In the present study, we established succinylation and acetylation databases in S. aureus XN108, a VISA strain with a high level of multidrug resistance (29). A total of 3,260 succinylation sites in 799 proteins and 7,935 Kac sites from 1,710 proteins were detected in XN108-WT strain. The numbers of Ksucc and Kac sites reported in this study were greater than those identified previously (27, 28), partially because of the benefit of the development and improvement of detection methodology. Another reason might be that different S. aureus strains with different backgrounds were used. The functional annotation revealed that the succinylated and acetylated proteins were closely related to a wide range of metabolic pathways, including TCA cycle, amino acid metabolism, and cell wall biogenesis. Several other bioinformatics analyses, including PPI and sequence motif, were also performed, and the results were highly consistent with others’ findings that Ksucc and Kac modifications are extensively spread in the regulatory networks of life (39). Therefore, protein succinylation and acetylation have important biological functions because of their universal existence in different organisms and in complicated regulatory networks (40).

The coexistence of multiple types of PTMs in one organism has been reported (35, 40). Some PTMs may even occur on the same residue of different protein copies, which is termed PTM cross talk, and synergistically regulate biological activities. The interactions between Ksucc and Kac have been identified in several bacteria, including E. coli (34, 35), M. tuberculosis (22), P. aeruginosa (30), and A. hydrophila (21). We wondered how Ksucc and Kac interact with each other in S. aureus. By comparing the succinylome and acetylome data, we observed extensive overlap between Kac and Ksucc in VISA strain XN108. Among the 3,260 succinylation sites, 2,445 sites were modified by Kac at the same time, and only 815 sites were uniquely succinylated. Similar to the findings from other bacteria (30), most overlapping proteins in S. aureus were associated with central metabolic pathways, indicating that Kac and Ksucc work together to affect bacterial metabolisms. Interestingly, we noticed that Ksucc or Kac has its own preferences in specific biological pathways. For instance, proteins associated with peptidoglycan synthesis were more frequently succinylated than acetylation modification. Such results provided valuable information when certain bacterial phenotypes were studied. Campbell et al. presented an interconnection between autolysin (Atl) succinylation and β-lactam susceptibility by observing that Atl was the most succinylated protein in their study (28). We speculate that the enrichment of Ksucc modification on proteins involved in cell wall synthesis plays a significant role in the vancomycin resistance of VISA XN108, because thickened cell walls are common VISA features (41, 42). We will test this hypothesis in our ongoing work. Elucidating the role of succinylated molecules in vancomycin-resistance regulation during VISA formation will provide new targets for the control of VISA infections.

Most PTMs are enzyme-catalyzed processes (43, 44). Identification of corresponding enzymes can benefit the understanding of the biological roles of PTMs. Du et al. reported that Sirt5, an NAD-dependent deacetylase, is responsible for the desuccinylation process in mammalian cells (45). In 2013, Colak et al. demonstrated that CobB protein exhibits comparable desuccinylation and deacetylation activities in E. coli (34), suggesting that some PTM-associated enzymes may have dual function. Nevertheless, Weinert et al. announced in 2013 that CobB of E. coli is not a global regulator of succinylation (35), in contrast with the results presented by Colak et al. (34). The discrepancy might be caused by different E. coli strains used or culture conditions performed. To our knowledge, this work is the first time SaCobB has been identified as an enzyme with both desuccinylation and deacetylation activities in VISA. Both Ksucc and Kac levels were slightly increased after *cobB* gene deletion. Importantly, we observed that only a small part of the identified Ksucc and Kac proteins were affected by SaCobB. These data indicated that other desuccinylases and deacetylases may exist in S. aureus and need further exploration.

In conclusion, we identified the lysine succinylome and acetylome in VISA strain XN108. Most of the modified proteins were enriched in cell metabolism pathways. We observed frequent cross talk between Ksucc and Kac PTMs in XN108. In addition, we identified SaCobB as a bifunctional enzyme with deacetylation and desuccinylation activities in S. aureus. These findings not only help us fully understand the metabolic regulation mechanisms of S. aureus but also provide a new direction for further investigation of VISA strains from the perspective of PTMs.

## MATERIALS AND METHODS

### Bacterial strains and reagents.

The VISA strain XN108 was originally isolated from a burn patient of Southwest hospital and was used as backbone strain. The laboratory S. aureus strain RN4220 was a gift of Baolin Sun (University of Science and Technology of China). All S. aureus strains were cultured in tryptic soy broth (TSB; Oxoid, UK). E. coli strains DH5α were obtained commercially and cultured in Luria-Bertani (LB) medium (Oxoid, UK). Unless otherwise indicated, all strains were cultured at 37°C, or at 30°C when carrying temperature-sensitive plasmids. The anti-succinyllysine and anti-acetylation antibodies were purchased from Jingjie PTM BioLab, Inc., (PTM BIO, China), and the goat anti-mouse IgG-horseradish peroxidase (HRP) antibody was purchased from Abmart Inc. (China).

### Construction of the *cobB* gene mutant strain.

The Δ*cobB* mutant was constructed by homologous recombination method as previously described (46). Briefly, approximately 1,000-bp DNA fragments upstream and downstream of the *cobB* gene were cloned into a temperature-sensitive shuttle vector pBT2 to achieve plasmid pBT2-Δ*cobB* with E. coli DH5α. Next, the plasmid was transformed into S. aureus RN4220 strain for restriction modification and then transformed into VISA strain XN108. The Δ*cobB* mutant was obtained by culturing pBT2-Δ*cobB* plasmid-carried XN108 in TSB at 42 and 25°C in turn. The correct mutant was confirmed by PCR and DNA sequencing. All primers used are listed in Table S1.

### Proteomic extraction, trypsin digestion, and peptide enrichment.

Three biological repeats of S. aureus XN108 and XN108-Δ*cobB* were prepared. All six bacterial samples were grown in TSB overnight, and bacterial cells were harvested by centrifugation at 5,000 × *g* for 10 min at 4°C. Cell pellets were washed twice with ice-cold phosphate-buffered solution (PBS; pH 7.2), resuspended with lysis buffer (1% sodium dodecyl sulfate [SDS], 1% protease inhibitor, 3 μM trichostatin A [TSA], and 50 mM nicotinamide [NAM]), and then lysed by ultrasonication. After that, the lysate was centrifuged at 12,000 × *g* at 4°C for 10 min to remove the remaining debris. Finally, the supernatants were collected, and the protein concentration was determined by bicinchoninic acid (BCA) kit (Bio-Rad, USA).

Equal amounts of XN108 and XN108-Δ*cobB* protein samples were used for trypsin digestion. First, protein samples of different strains were adjusted to the same volume by adding lysis buffer. Then, one volume of cold acetone was added into protein samples, followed by mixing on vortex. The mixture was added with another four volumes of cold acetone and kept at −20°C for 2 h for protein precipitation. After centrifugation at 4,500 × *g* for 5 min, the supernatant was discarded, and the remaining precipitates were washed twice with cold acetone. After that, the air-dried precipitations were dissolved in 200 mM tetraethyl ammonium bromide (TEAB) for sonication and further added with trypsin for digestion overnight (the ratio of trypsin to protein was 1:50). The protein solution was reduced with 5 mM dithiothreitol (DTT) for 30 min at 56°C and alkylated with 11 mM iodoacetamide (IAA) for 15 min in darkness at room temperature.

The peptides were dissolved in IP buffer (100 mM NaCl, 1 mM EDTA, 50 mM Tris-HCl, and 0.5% NP-40, pH 8.0), and the supernatants were transferred to the prewashed succinylated resin (PTM-402, PTM Bio) or acetylated resin (PTM-104, PTM Bio) at 4°C overnight with gentle shaking. The resins were washed four times with IP buffer and twice with double-distilled water (ddH_2_O). The resin-bound peptides were eluted three times by using 0.1% trifluoroacetic acid (TFA) and vacuum-dried. Finally, the obtained peptides were desalted with C18 ZipTips (Millipore) according to the manufacturer’s instructions and were analyzed by LC-MS/MS.

### LC-MS/MS analysis and database search.

The peptides were dissolved in liquid chromatography solvent A (0.1% formic acid and 2% acetonitrile) and a gradient of solvent B (0.1% formic acid and 100% acetonitrile) for separation by using NanoElute (Bruker Daltonics, USA) with ReproSil-Pur Basic C18 column (1.9 μm, 100 μm, 25 cm). The gradient of solvent B was set as following: increased from 6 to 22% for 0 to 44 min, from 22 to 35% for 44 to 56 min, 35 to 80% for 56 to 58 min, and at 80% for 58 to 60 min. All increments were performed at a constant flow rate of 450.00 nL/min on a NanoElute system. The peptides were separated by NanoElute and were ionized by injecting into the capillary ion source. Then, the peptide segments were analyzed by TIMS-TOF Pro mass spectrometry (Bruker Daltonics, USA). The electrospray voltage was set at 2.0 kV. The parent ion of the peptide segments and their secondary fragments were detected and analyzed by using high resolution time-of-flight (TOF). The MS scan range was 100 to 1,700 for full scan, and the parallel cumulative serial fragmentation (PASEF) mode was used for data acquisition. Secondary MS scan with the charge of the parent ions in the range of 0 to 5 were collected in PASEF mode (peptide charge of 0 to 5 is set as the default parameter by the mass spectrometer NanoElute [Bruker Daltonics, USA], and it can automatically exclude +1 ion interference), followed by 10 MS/MS scans with 30-s dynamic exclusion.

The resulting MS/MS data were processed using Maxquant 1.6.6.0. Tandem mass spectra were searched against the S. aureus XN108 (2,992 sequences) database concatenated with a reverse decoy database. Trypsin/P was specified as cleavage enzyme allowing up to four missing cleavages, seven modifications per peptide, and five charges. The mass tolerance for precursor ions was set as 10 ppm in the first search and 20 ppm in the main search, and the mass tolerance for fragment ions was set as 0.02 Da. Carbamidomethylation on cysteine was set as a fixed modification. Acetylation modification on the protein N terminus, oxidation on methionine, and succinylation and acetylation on lysine were specified as variable modifications. Label-free quantification (LFQ) was used for a quantitative method. The false discovery rate (FDR) thresholds for protein, peptide, and modification sites were set to 1%.

### Immunoprecipitation (IP) experiment.

The IP test was performed to pull down the MurA protein, which was detected to be succinylated in our MS studies. Briefly, the S. aureus strains of interest were cultured in TSB medium overnight. Bacterial cells were harvested by centrifugation and lysed by ultrasonication, and then the supernatant was collected after centrifugation. The remaining cell lysate was incubated and immunoprecipitated with rabbit anti-MurA polyclonal antibody (produced in our laboratory) and 50 μL of SPA-coated beads (Santa Cruz Biotechnology, USA) at 4°C overnight with shaking. The serum of an unimmunized rabbit served as isotype control. In the next day, the immune complexes were washed with sterilized PBS five times, and the pellets were resuspended in 1× SDS sample buffer followed by boiling for 5 min. Then, the precipitated proteins were collected by centrifugation and a Western blot by using mouse anti-succinyllysine MAb to confirm the succinylation of MurA protein.

### Bioinformatics analysis.

**(i) Annotation methods.** GO annotation proteome was derived from the UniProt-GOA database (http://www.ebi.ac.uk/GOA/) and was used to identify succinylated or acetylated proteins, first converting identified protein ID to the UniProt ID and then mapping to GO IDs by protein ID. If some identified proteins were not annotated by UniProt-GOA database, the InterProScan software would be used to annotate the protein’s GO function based on the protein sequence alignment method. Then, all identified proteins were classified by GO annotation into three categories: biological process, cellular component, and molecular function. The domain functional description of identified proteins was annotated by using InterProScan software based on a protein sequence alignment method, and the InterPro domain database (http://www.ebi.ac.uk/interpro/) was used. The KEGG database was used to annotate protein pathway. The KEGG online service tool KAAS was used to annotated protein’s KEGG database description, followed by mapping the annotation results on the KEGG pathway database using KEGG mapper. The subcellular localization prediction software WoLF PSORT was used, and the subcellular structures of prokaryotes were assigned using CELLO software. Motif-x and MoMo were used for analysis of the amino acids, which are overrepresented or underrepresented at the −10 to +10 position adjacent to a succinylation site (or an acetylation site).

**(ii) Functional enrichment.** For each GO annotation category, a two-tailed Fisher’s exact test was employed to test the enrichment of the differentially expressed protein against all identified proteins. The GO with a corrected *P* value of <0.05 is considered significant. KEGG database was used to identify enriched pathways by a two-tailed Fisher’s exact test to test the enrichment of the differentially expressed protein against all identified proteins. The pathway with a corrected *P* value of <0.05 was considered significant.

**(iii) Enrichment-based clustering.** All of the categories obtained after enrichment along with their *P* values were collated, and then those categories that were enriched in at least one of the clusters with *P* values <0.05 were filtered. This filtered *P*-value matrix was transformed by the function *x* = −log_10_ (*P* value). Finally, these *x* values were *z*-transformed for each functional category. These *z* scores were then clustered by one-way hierarchical clustering (Euclidean distance and average linkage clustering) in the Genesis.

**(iv) Protein-protein interaction network.** PPI networks were analyzed using the STRING database, version 11.0. Only interactions between the proteins belonging to the searched data set were selected, thereby excluding external candidates. STRING defines a metric called “confidence score” to define interaction confidence. All interactions that had a confidence score of ≥0.7 (high confidence) were fetched, and the interaction work from STRING was achieved and visualized with Cytoscape software.

### Quantification analysis.

The raw LC-MS data sets were first searched against database and converted into matrices containing reporter intensity of peptides across samples. The relative quantitative value of each modified peptide was then calculated based on intensity information by the following steps. First, the intensities of modified peptides (*I*) were centralized and transformed into relative quantitative values (*U*) of modified peptides in each sample. The formula is listed as follows: *R_ij_* = *I_ij_*/mean (*I_j_*) (where *i* denotes the sample, and *j* denotes the modified peptide). Second, the ratio of the average value between the two samples is calculated. The ratio is used as the final quantitation. For normalization to succinylated (or acetylated) peptides, the naked intensities of succinylated peptides (or acetylated peptides) were first measured and then were divided by the corresponding protein intensities. If both proteomics and PTM profiling were conducted on the same cohort, the relative quantitative value of the modified peptide is divided by the relative quantitative value of corresponding protein to remove the influence from protein expression of modifications. Finally, the significance *P* value of the difference modification is calculated.

The relative quantitative values of each sample were set as log_2_ transform (to make the data conform to the normal distribution), and then *P* value was calculated by the two-sample two-tailed *t* test method. Sites with XN108-Δ*cobB*/XN108-WT ratios of >1.5 and *P* values of <0.05 were considered upregulated succinylation (or acetylation). Sites with XN108-Δ*cobB*/XN108-WT ratios of <1/1.5 and *P* values of <0.05 were considered downregulated succinylation (or acetylation).

### Western blot.

Equal amounts of protein samples from different bacteria strains were separated by 12% SDS-polyacrylamide gel electrophoresis (SDS-PAGE), and the proteins were transferred to polyvinylidene fluoride (PVDF) membranes. Anti-succinyllysine mouse MAb (PTM Bio) or anti-acetyllysine mouse MAb (PTM Bio) at a dilution of 1:1,000 was used as the first antibody, respectively. Secondary antibody conjugated to horseradish peroxidase (Beyotime, China) was diluted by 1:5,000. The images were visualized with a chemiluminescent detection imager (Bio-Rad, USA).

### Statistical analysis.

Statistical analysis of results was carried out using GraphPad Prism 5. Unpaired two-tailed Student’s *t* test was used to treat samples between two groups, and one-way analysis of variance (ANOVA) was used for testing multiple groups. Each experiment was carried out at least three times. The results are presented as mean ± standard deviations (SDs), and a *P* value of less than 0.05 was considered statistically significant (*, *P* < 0.05; **, *P* < 0.01; ***, *P* < 0.001; ns, no significance).

### Data availability.

The raw data and annotated MS spectra are available in the proteomics repository PRIDE with ProteomeXchange data set accession number of PXD033341.

## Supplementary Material

Reviewer comments
