# Supplementary material for: Identification of Lysine Succinylome and Acetylome in the Vancomycin-Intermediate Staphylococcus aureus XN108
Source: Microbiol Spectr. 2022 Nov 14;10(6):e03481-22. doi: 10.1128/spectrum.03481-22 (PMC9769639; doi:10.1128/spectrum.03481-22)
Supplement: Supplemental file 9 — Supplemental material. Download spectrum.03481-22-s0009.pdf, PDF file, 0.9 MB [file spectrum.03481-22-s0009.pdf]

**Supplemental Material**

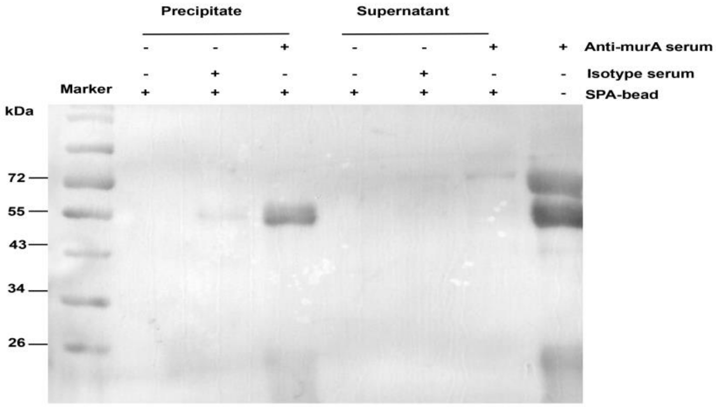


**Fig. S1** Validation of MurA protein succinylation in XN108 by IP experiment. Rabbit anti-MurA antibody plus SPA-coated beads were used to pull down the target proteins, SPA-coated beads with or without normal rabbit serum served as control. The precipitated proteins were collected and a Western blot by using mouse anti-succinyllysine mAb was performed to confirm the succinylation of MurA.

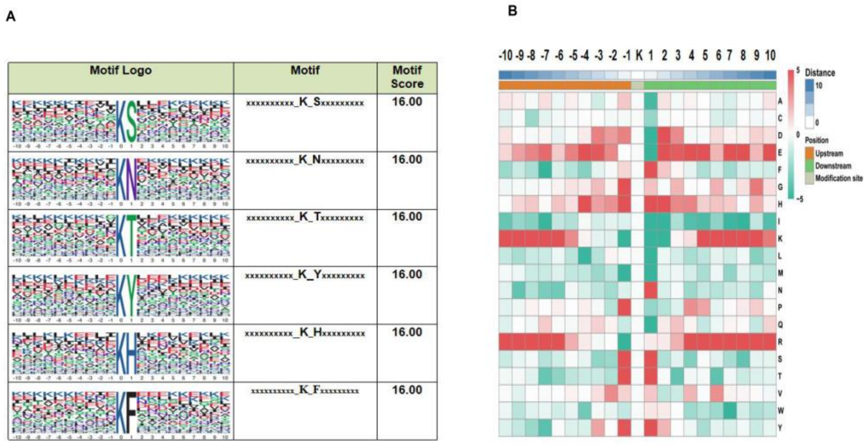


**Fig. S2** Motif analysis of identified lysine-acetylated sequences. (A) Kac sequences were analyzed using the Motif-x tool. The top six lysine acetylation motifs are shown. (B) Heatmap shows the frequency of different amino acid residues around acetylated lysine residues in *S. aureus.*

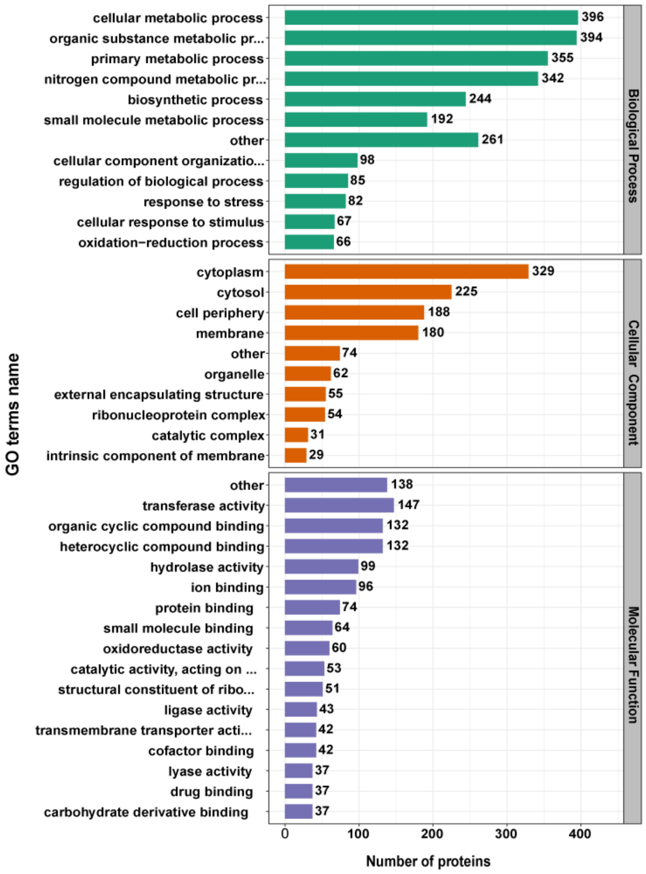


**Fig. S3** GO analysis of Kac proteins in VISA XN108. GO analysis for the biological processes, cellular component, and molecular function of the identified acetylated proteins in XN108.

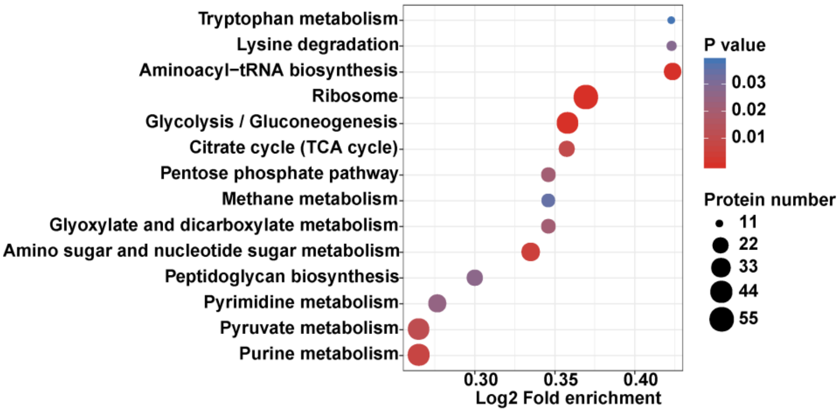


**Fig. S4** KEGG pathway analysis of Kac proteins in VISA XN108. KEGG categories of the acetylated proteins in XN108.

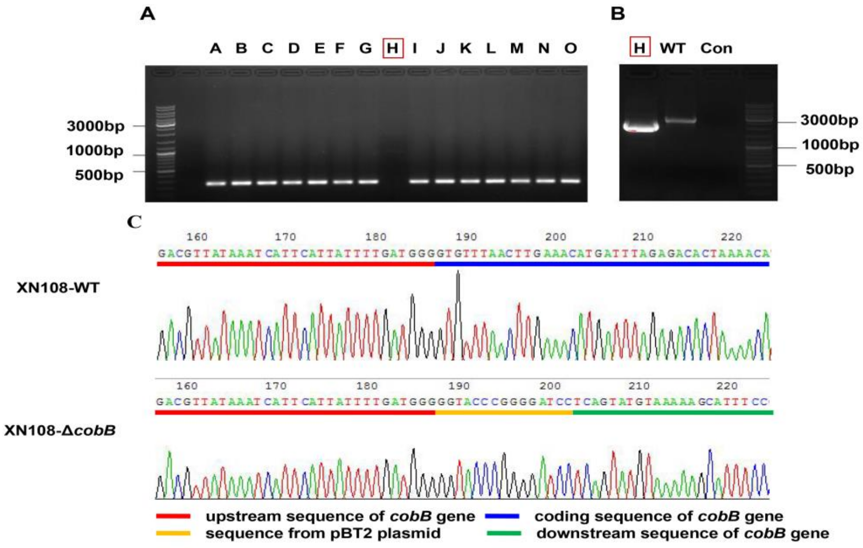


**Fig. S5** Construction and identification of XN108-Δ*cobB* mutant. PCR experiments by using primer pairs located in *cobB* gene coding sequence (A) or primer pairs flanking *cobB* gene (B) were performed. The *cobB* gene was successfully deleted in H clone. Gene sequencing was further carried out to confirm the construction of XN108-Δ*cobB* mutant strain (C). All primers are showed in TABLE S1.

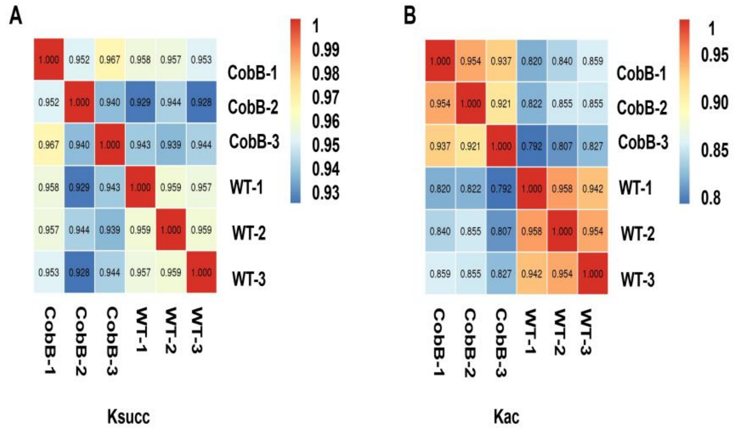


**Fig. S6** Pearson correlation coefficient analysis between XN108-Δ*cobB* and XN108-WT. Correlation of the protein intensity (log10 scale) among three biological replicates of the XN108-WT (WT-1, WT-2, and WT-3) and XN108-*cobB* mutant (CobB-1, CobB-2, and CobB-3). (A) Pearson correlation coefficient of the succinylation samples. (B) Pearson correlation coefficient of the acetylation samples.

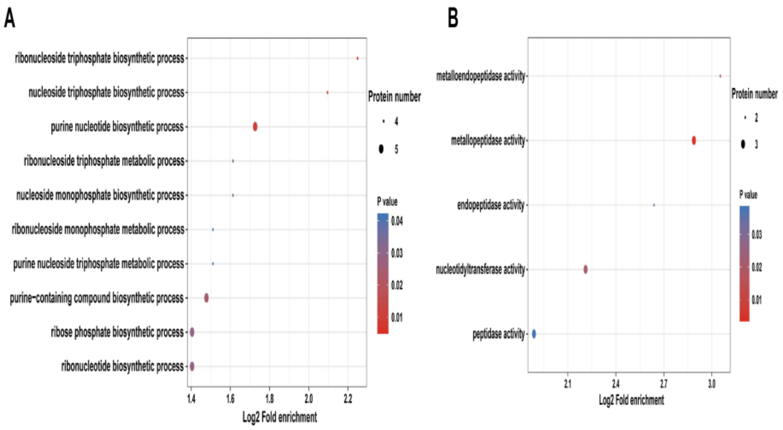


**Fig. S7** GO enrichment analysis of differently expressed Ksucc proteins. GO analysis for the biological processes (A) and molecular function (B) of the identified succinylation proteins regulated by SaCobB.

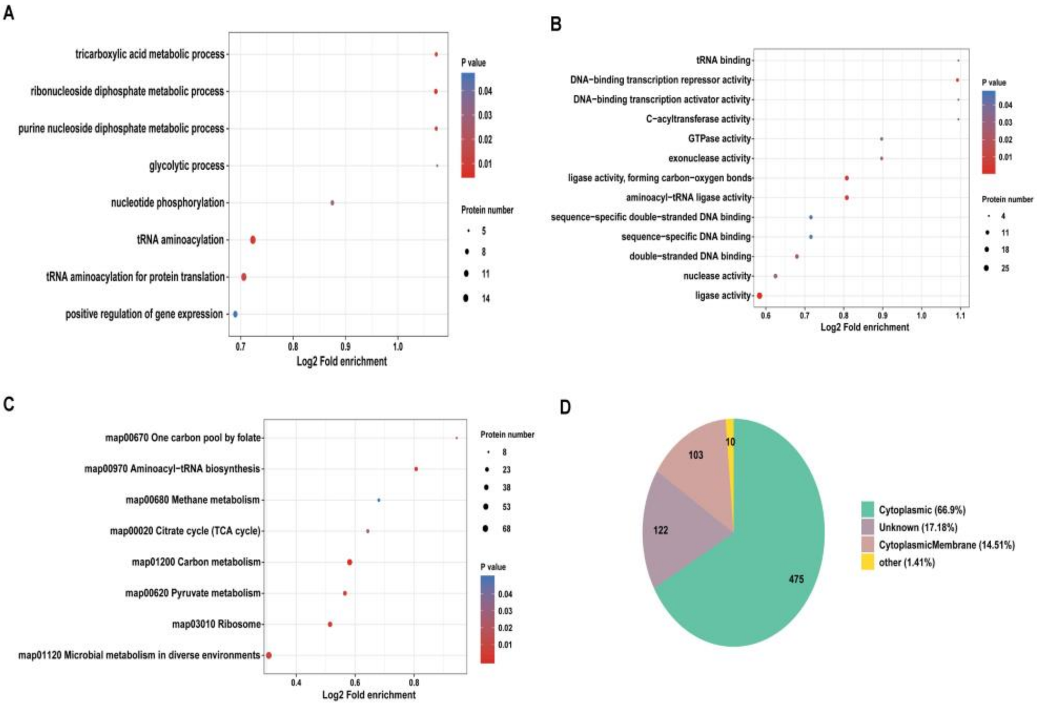


**Fig. S8** GO enrichment and KEGG pathway analysis of differently expressed acetylated proteins regulated by SaCobB. GO analysis for the biological processes (A) and molecular function (B) of the identified acetylated proteins regulated by SaCobB. (C) KEGG categories of acetylated proteins regulated by SaCobB. (D) Subcellular localization of the identified acetylated proteins regulated by SaCobB from GO analysis.

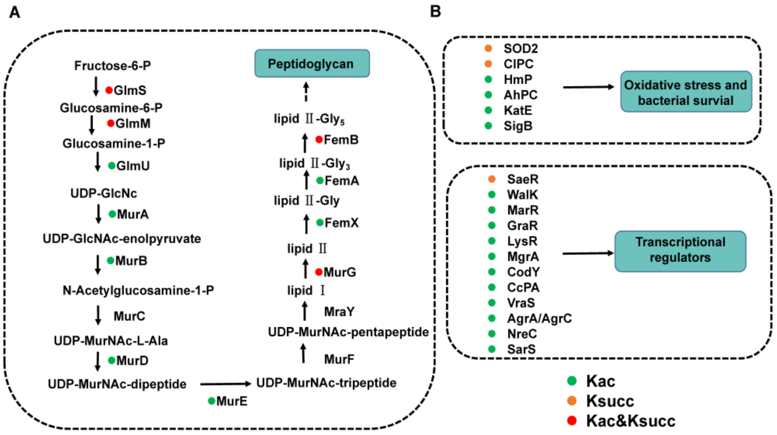


**Fig. S9** Representing regulatory role of SaCobB in central metabolic enzymes that are succinylated or acetylated alteration in VISA XN108. (A) The succinylation or acetylation altered enzymes for the peptidoglycan synthesis process. (B) The succinylation or acetylation changed enzymes associated with oxidative stress and bacterial survival, and transcriptional regulators. The green, yellow and red circle dots represent enzymes modified by unique acetylated alteration, unique succinylated alteration, both succinylated and acetylated alterations, respectively.

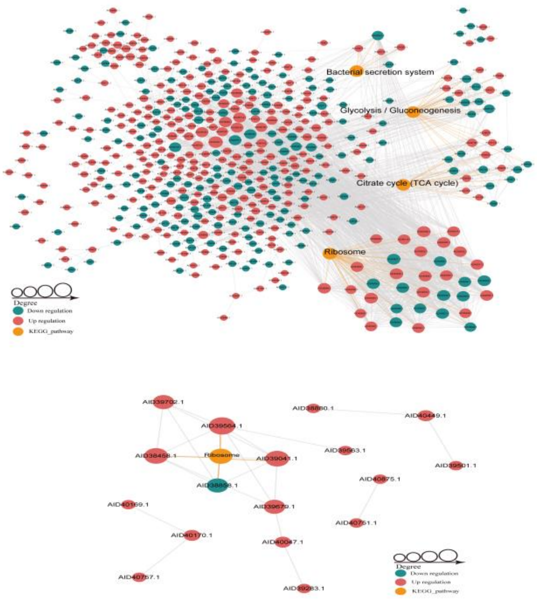


**Fig. S10** KEGG combined with PPI analysis of altering acetylated proteins and succinylated proteins regulated by SaCobB. Analysis of functional interactions of SaCobB regulated acetylated (A) and succinylated (B) and proteins using STRING and Cytoscape with FDR<0.05. The blue and red circle dots represent the differentially expressed proteins (the blue for the downregulated proteins, the red for the upregulated proteins), the orange represent KEGG pathway. The protein IDs of the nodes in the network are shown in Data sheet S9.

**Table S1.** Primers used in this study

| primer name | Sequences (5'-3') | Description |
|---|---|---|
| cobBL-F | CGCGAATTCTTCGTTTAACTGACAAAATG | For amplification of upstream region of *cobB* gene, and the amplicon to construct pBT2-*cobB1* |
| cobBL-R | CGCGGTACCCCCATCAAAATAATGAATG | |
| cobBR-F | CGCGGATCCTCAGTATGTAAAAAGCAT | For amplification of downstream region of *cobB* gene, and the amplicon to construct pBT2-*cobB2* |
| cobBR-R | CGCGTCGACAGCTTCTTTAACTAAAGAA | |
| cobBin-F | CGTATTACATTTTTTACAGGTGCTG | For amplification part of *cobB* gene to confirm the double crossover event |
| cobBin-R | TATTAATCCTGCGGCAGGTT | |
| cobBouter-F | TTCAATATATGTTTTAATCGTTTC | For amplification of upstream and downstream regions of *cobB* gene to confirm the double crossover event |
| cobBouter-R | ACCAAAAACATTAATAAAAGTTTG | |

**Supplementary Data sheets**

**Data Set S1** Identified succinylated peptides and proteins in XN108.

**Data Set S2** Identified acetylated peptides and proteins in XN108.

**Data Set S3** Common sites in protein succinlytion and acetylation in XN108.

**Data Set S4** All identified succinylated peptides and proteins in XN108-WT and XN108-Δ*cobB.*

**Data Set S5** Upregulated and downregulated succinylated sites of proteins in XN108-Δ*cobB.*

**Data Set S6** All identified acetylated peptides and proteins in XN108-WT and XN108-Δ*cobB.*

**Data Set S7** Upregulated and downregulated acetylated sites of proteins in XN108-Δ*cobB.*

**Data Set S8** Identified proteins involved in virulence and resistance.
