# Supplementary material for: Identification of Lysine Succinylome and Acetylome in the Vancomycin-Intermediate Staphylococcus aureus XN108
Source: Microbiol Spectr. 2022 Nov 14;10(6):e03481-22. doi: 10.1128/spectrum.03481-22 (PMC9769639; doi:10.1128/spectrum.03481-22)
Supplement: Reviewer comments [file reviewer-comments.pdf]

AMERICAN SOCIETY FOR MICROBIOLOGY | Microbiology Spectrum

Identification of lysine succinylome and acetylome in the vancomycin-intermediate Staphylococcus aureus XN108

Xiancai Rao, Li Tan, Yi Yang, Weilong Shang, Zhen Hu, Huagang Peng, Shu Li, and Xiaomei Hu

Corresponding Author(s): Xiancai Rao, Army Medical University

Review Timeline:

Submission Date:	September 7, 2022
Accepted:	October 22, 2022

Editor: Mariola Edelmann

Reviewer(s): Disclosure of reviewer identity is with reference to reviewer comments included in decision letter(s). The following individuals involved in review of your submission have agreed to reveal their identity: Fangyou Yu (Reviewer #1); Jianping Xie (Reviewer #2)

Transaction Report:

(Note: With the exception of the correction of typographical or spelling errors that could be a source of ambiguity, letters and reports are not edited. The original formatting of letters and referee reports may not be reflected in this compilation.)

DOI: https://doi.org/10.1128/spectrum.03481-22

October 22, 2022

Prof. Xiancai Rao
Army Medical University
Department of Microbiology, College of Basic Medical Sciences
Gaotanyan Road 30, Shapingba District, Chongqing, China.
Chongqing 400038
China

Re: Spectrum03481-22 (Identification of lysine succinylome and acetylome in the vancomycin-intermediate Staphylococcus aureus XN108)

Dear Prof. Xiancai Rao:

Your manuscript has been accepted, and I am forwarding it to the ASM Journals Department for publication. You will be notified when your proofs are ready to be viewed.

The ASM Journals program strives for constant improvement in our submission and publication process. Please tell us how we can improve your experience by taking this quick Author Survey.

As an open-access publication, Spectrum receives no financial support from paid subscriptions and depends on authors' prompt payment of publication fees as soon as their articles are accepted. You will be contacted separately about payment when the proofs are issued; please follow the instructions in that e-mail. Arrangements for payment must be made before your article is published. For a complete list of **Publication Fees**, including supplemental material costs, please visit ourwebsite.

ASM policy requires that data be available to the public upon online posting of the article, so please verify all links to sequence records, if present, and make sure that each number retrieves the full record of the data. If a new accession number is not linked or a link is broken, provide production staff with the correct URL for the record. If the accession numbers for new data are not publicly accessible before the expected online posting of the article, publication of your article may be delayed; please contact the ASM production staff immediately with the expected release date.

Corresponding authors may join or renew ASM membership to obtain discounts on publication fees. Need to upgrade your membership level? Please contact Customer Service at Service@asmusa.org.

Thank you for submitting your paper to Spectrum.

Sincerely,

Mariola Edelmann
Editor, Microbiology Spectrum

Journals Department
American Society for Microbiology
1752 N St., NW
Washington, DC 20036
E-mail: spectrum@asmusa.org

Supplemental Dataset: Accept
Supplemental Dataset: Accept
Supplemental Dataset: Accept
Supplemental Dataset: Accept
Supplemental Dataset: Accept
Supplemental Dataset: Accept
Supplemental Dataset: Accept
Supplemental Dataset: Accept
